# A New Cytotoxicity Assay for Brevetoxins Using Fluorescence Microscopy

**DOI:** 10.3390/md12094868

**Published:** 2014-09-23

**Authors:** Jennifer R. McCall, Elizabeth A. Elliott, Andrea J. Bourdelais

**Affiliations:** NCW Center for Marine Science, 5600 Marvin K Moss Lane, Wilmington, NC 28409, USA; E-Mails: elliotte@uncw.edu (E.A.E.); bourdelaisa@uncw.edu (A.J.B.)

**Keywords:** brevetoxins, cytotoxicity assay, triphasic response, hormesis, Neuro-2A, SJCRH30

## Abstract

Brevetoxins are a family of ladder-framed polyether toxins produced during blooms of the marine dinoflagellate, *Karenia brevis*. Consumption of shellfish or finfish exposed to brevetoxins can lead to the development of neurotoxic shellfish poisoning. The toxic effects of brevetoxins are believed to be due to the activation of voltage-sensitive sodium channels in cell membranes. The traditional cytotoxicity assay for detection of brevetoxins uses the Neuro-2A cell line, which must first be treated with the neurotoxins, ouabain and veratridine, in order to become sensitive to brevetoxins. In this study, we demonstrate several drawbacks of the Neuro-2A assay, which include variability for the EC_50_ values for brevetoxin and non-linear triphasic dose response curves. Ouabain/veratridine-treated Neuro-2A cells do not show a typical sigmoidal dose response curve in response to brevetoxin, but rather, have a polynomial shaped curve, which makes calculating EC_50_ values highly variable. We describe a new fluorescence live cell imaging model, which allows for accurate calculation of cytotoxicity via nuclear staining and additional measurement of other viability parameters depending on which aspect of the cell is stained. In addition, the SJCRH30 cell line shows promise as an alternative to Neuro-2A cells for testing brevetoxins without the need for ouabain and veratridine.

## 1. Introduction

Brevetoxins (PbTxs) are a family of ladder frame polyether neurotoxins produced during blooms of the marine dinoflagellate, *Karenia brevis* [1]. In the United States, *K*.* brevis* blooms are frequent, nearly annual occurrences off the coast of Florida, but also occur in North Carolina and other areas in the Gulf of Mexico. The PbTxs produced during these blooms are potent neurotoxins that are known to cause massive fish kills and a large number of mortalities in seabirds, sea turtles and marine mammals [2,3,4,5]. The toxic effects of PbTxs are due to binding to and persistent activation of voltage sensitive sodium channels (VSSCs) [6,7,8]. Humans can become exposed to PbTxs if they consume contaminated shellfish or finfish, which may cause the development neurotoxic shellfish poisoning (NSP), or exposure can occur through sea spray, which may cause respiratory dysfunction in beach visitors. Symptoms of NSP include gastroenteritis, sensory abnormalities, cranial nerve dysfunction and other neurotoxic effects. In severe cases, victims may require emergency room treatment to prevent respiratory failure [9,10]. The most common symptoms of exposure to aerosolized PbTxs are eye irritation and acute respiratory irritation of both the upper and lower respiratory tracts, including nasal congestion, throat irritation, cough, chest tightness, wheezing and shortness of breath [11]. People who are particularly susceptible, such as asthmatics and those with COPD, may develop more severe symptoms and require hospitalization following exposure to inhaled PbTxs [12,13]. PbTxs are not destroyed by cleaning, cooking or freezing of fish and cannot be detected by taste or smell, making accurate detection of toxins during *K*.* brevis* blooms paramount for consumer safety.

The traditional method for detecting the presence of PbTxs in a sample has been the mouse bioassay, which is the official regulatory method in most countries for determining toxin levels. The mouse bioassay is a whole animal test in which an extracted sample is injected into the peritoneal cavity of a mouse and time to death is monitored. Results are reported as mouse units (MU) per 100 g of shellfish, where one mouse unit is the amount of crude toxin that on average will kill 50% of mice in 15.5 h [14]. The current U.S. guidelines limit PbTx concentrations in shellfish to 20 MU per 100 g of shellfish, where 1 MU is equivalent to 4 μg PbTx-2. This is equivalent to 0.8 ppm of PbTx-2 [15]. However, there are several drawbacks to the mouse bioassay for use as a regular screening mechanism for NSP. These include long time requirements (2–24 h to measurable death and then up to seven days of post-injection observations for no toxicity), high cost ($100 per test), low specificity (any toxic compound in the sample can kill the mouse), low sample throughput, due to labor-intensive requirements, high variability (due to differences in mouse strain, age and weight) and high animal usage (one animal per test) [14,15,16,17,18].

In an effort to move away from the cumbersome and expensive mouse bioassay, a cytotoxicity assay was developed using a mouse neuroblastoma (Neuro-2A) cell line. In the original assay developed for testing saxitoxins and tetrodotoxin, Neuro-2A cells were treated with veratridine (a VSSC activator) and ouabain (a sodium/potassium pump inhibitor), resulting in sodium influx into the cell and subsequent cell death. Saxitoxins and tetrodotoxin could be detected by their ability to prevent cell death, presumably because of binding to and blocking VSSCs, thus inhibiting sodium influx into the cell [19,20]. Manger and colleagues expanded this test to PbTxs with the observation that PbTx treatment sped up cell death, likely due to synergistic activity between veratridine and PbTxs, both of which bind to and activate VSSCs [21,22]. The development of this assay has allowed for the study of the cytotoxicity of various PbTx analogs and extracts, as well as extracts from other *Karenia* species [23,24,25,26,27]. Some investigators have found that the Neuro-2A assay is not as sensitive for detecting PbTxs in samples as other assays, such as LC-MS or receptor binding assays [24,28].

A survey of the literature indicates great variability in methods associated with the Neuro-2A cytotoxicity assay. The ouabain concentration was typically 500 μM, but the veratridine concentration ranged from 5 to 50 μM. The time from treatment to measurement varied from 12 to 24 h, and cell seeded density ranged 6000–100,000 [19,20,21,22,23,24,25,26,27,28,29,30]. The most commonly used method for determining cell death was the MTT colorimetric assay for determining cell viability, in which metabolically active cells reduce the MTT tetrazolium compound to form a blue color. More intense color indicates greater cell viability, and high throughput can be achieved by using a multiwell plate format [21]. It was our intent to develop an improved cytotoxicity assay for PbTxs using a human, rather than rodent, cell line with a more direct detection method that counts individual live cells rather than the MTT assay, which only measures cytotoxicity indirectly. In addition, it was our goal to develop a sensitive cytotoxicity assay using a cell line that did not require the use of ouabain and veratridine to pre-sensitize cells to PbTx toxicity, which likely causes unknown side-effects and uncontrolled variability. To that end, we surveyed seven alternative cell lines for sensitivity to ouabain, veratridine and PbTxs. This report describes the development of a new cytotoxicity assay using fluorescence detection of cytotoxicity using a direct measurement of nucleated cells.

## 2. Results and Discussion

### 2.1. Cell Line Survey for Brevetoxin Cytotoxicity

**Figure 1:**
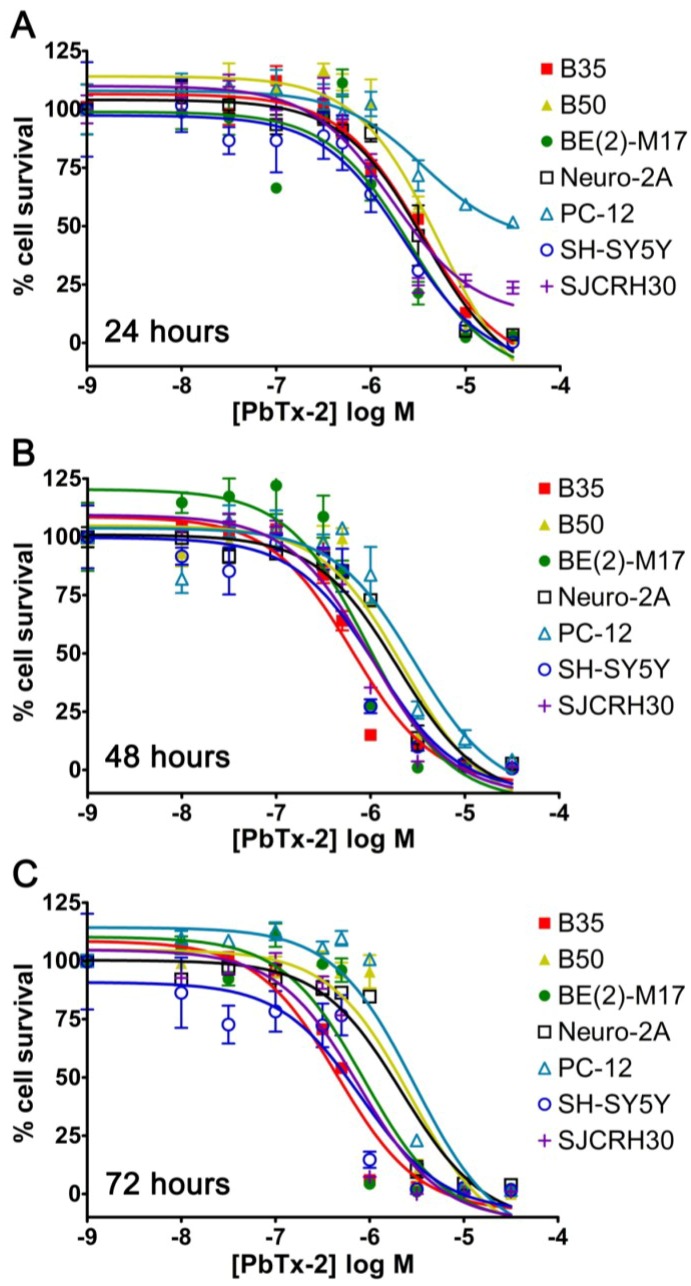
Sigmoidal dose response curves to PbTx-2 from seven cell lines at (**A**) 24 h, (**B**) 48 h and (**C**) 72 h post-treatment (*n =* 4). Each point represents the mean ± standard deviation for *n =* 4 replicates.

In order to determine alternative cell line candidates for a PbTx cytotoxicity assay, several cell lines were screened in the presence and absence of ouabain (500 μM) and veratridine (25 μM) for toxicity to PbTx-2. The choices of concentrations were based on previous literature: 500 μM of ouabain was the typical concentration used, and 25 μM of veratridine was the approximate middle of the range (5–50 μM) used [19,20,21,22,23,24,25,26,27,28,29,30]. Cell lines were chosen in the attempt to find a human alternative to the rodent neuroblastoma (Neuro-2A) cell line and to survey other neural tissue-derived cells to determine if they have greater sensitivity to PbTxs without the use of ouabain and veratridine. Cytotoxicity was measured by staining and counting nuclei with the Image Xpress Micro, which is a live cell imager. As seen in Table 1 and Figure 1, sensitivity to PbTx-2 without ouabain and veratridine was in the micromolar range for all cell lines after 24 h. Sensitivity increased in most cell lines following 48 h of treatment. EC_50_ values (Table 1) were calculated from four well replicates in Figure 1.

**Table 1:** Cell line survey for brevetoxin 2 (PbTx-2) cytotoxicity. EC_50_ values are calculated from the curves generated in Figure 1.

Cell Line	Cell Type	Seeded Density	24 h EC_50_	48 h EC_50_	72 h EC_50_
B35	Rat neuronal neuroblastoma	5000	3.39 × 10^−6^ M	6.40 × 10^−7^ M	4.57 × 10^−7^ M
B50	Rat neuronal neuroblastoma	5000	5.24 × 10^−6^ M	2.22 × 10^−6^ M	2.70 × 10^−6^ M
BE(2)-M17	Human neuroblastoma	5000	2.60 × 10^−6^ M	9.40 × 10^−^^7^ M	8.77 × 10^−^^7^ M
Neuro-2A	Mouse neuronal neuroblastoma	5000	3.66 × 10^−6^ M	1.94 × 10^−6^ M	2.18 × 10^−6^ M
PC-12	Rat adrenal pheochromocytoma	10,000	3.60 × 10^−6^ M	2.92 × 10^−6^ M	2.99 × 10^−6^ M
SH-SY5Y	Human bone marrow neuroblastoma	5000	2.26 × 10^−6^ M	1.14 × 10^−6^ M	8.11 × 10^−^^7^ M
SJCRH30	Human muscle rhabdomyosarcoma	5000	1.70 × 10^−6^ M	8.61 × 10^−^^7^ M	7.69 × 10^−^^7^ M

The cell lines greatly differed with co-treatment of ouabain (500 μM) and veratridine (25 μM). As seen in Table 2, ouabain and veratridine in the ab sence of PbTx-2 resulted in nearly complete cytotoxicity in all human cell lines within 24 h. Ouabain (at all concentrations) and veratridine above 5 μM has previously been reported to be cytotoxic in the human embryonic kidney cell line, HEK-293 [31]. For the rodent cell lines, B35, B50 and Neuro-2A, roughly half of all cells died following 24 h after treatment with ouabain and veratridine and exhibited little to no cell growth over the next 72 h. Nearly all Neuro-2A cells were dead by 72 h after treatment with ouabain and veratridine (Table 2). Interestingly, the rat PC12 cell line did not show much initial sensitivity to ouabain and veratridine, and it was the only cell line to grow to greater cell numbers after 72 h with ouabain and veratridine treatment, as compared to 24 h without ouabain and veratridine (Table 2).

**Table 2:** Cell line sensitivity to ouabain (500 μM) and veratridine (25 μM).

Cell Line	Time Point	Cell Count without Ouabain/Veratridine	Cell Count with Ouabain/Veratridine	Percentage Knockdown (of 24 h)
B35	24 h	199.2	105.4	47%
B35	48 h	420.9	120.3	40%
B35	72 h	1020.3	153.2	23%
B50	24 h	201.6	106.9	47%
B50	48 h	541.1	122.8	39%
B50	72 h	820.6	158.4	21%
BE(2)-M17	24 h	164.3	5.5	97%
BE(2)-M17	48 h	296.0	3.9	98%
BE(2)-M17	72 h	696.2	7.8	95%
Neuro-2A	24 h	532.0	270.8	49%
Neuro-2A	48 h	722.8	105.2	80%
Neuro-2A	72 h	766.8	48.3	91%
PC-12	24 h	334.2	275.3	18%
PC-12	48 h	540.1	317.4	5%
PC-12	72 h	927.5	602.2	−80%
SH-SY5Y	24 h	73.5	8.2	89%
SH-SY5Y	48 h	71.0	9.7	87%
SH-SY5Y	72 h	62.4	2.7	96%
SJCRH30	24 h	208.0	1.8	99%
SJCRH30	48 h	306.0	0.4	100%
SJCRH30	72 h	570.1	0.1	100%

Previous studies had stated that ouabain (up to 2 mM) and veratridine (up to 0.1 mM) alone were not toxic to Neuro-2A cells [19]. In our present study, dose response curves of ouabain (up to 500 μM) or veratridine (up to 50 μM) alone confirmed little toxicity to Neuro-2A after 24 h (80% and 100% cell survival, respectively). However, this declines by 48 h to 52% and 75% cell survival for the highest doses of ouabain and veratridine, respectively. When added together, 500 μM ouabain and 50 μM veratridine yielded 82% cell survival after 24 h and 50% cell survival after 48 h, which is not significantly different from the results obtained with ouabain alone. When only 25 μM veratridine were added to 500 μM ouabain, cell survival was 79% after 24 h and 58% cell survival after 48 h, which was not significantly different from the values obtained with 500 μM ouabain and 50 μM veratridine or from 500 μM ouabain alone.

**Figure 2:**
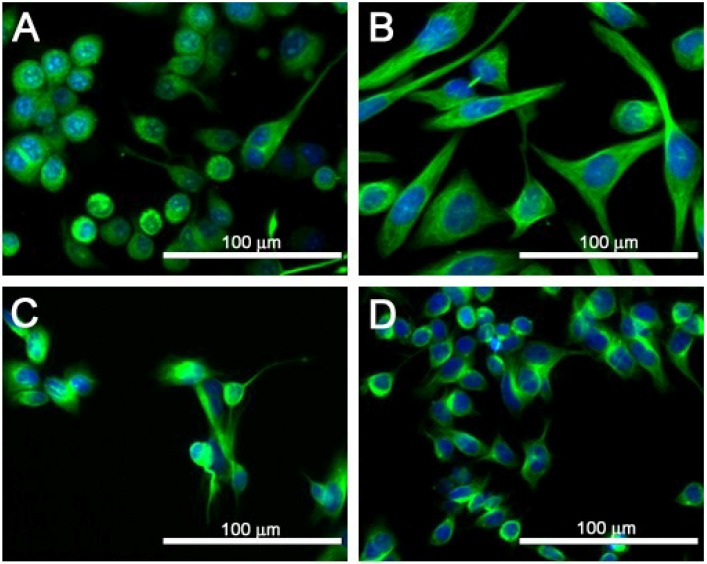
Images of (**A**) Neuro-2A; (**B**) SJCHR30; (**C**) SH-SY5Y and (**D**) BE(2)-M17 cell lines. Cells were stained with Hoechst 33342 nuclear dye (blue) and Tubulin Tracker (green). The scale bar is at 100 μm.

The three human cell lines surveyed were examined for cell morphology and compared to the Neuro-2A cell line. As shown in Figure 2, SJCRH30 cells were large and spread out, as compared to the smaller BE(2)-M17 and SH-SY5Y cells, which allowed for greater counting efficiency for live cell imaging and allowed for easier measurement and visualization of morphological changes associated with toxin exposure. While all four cell lines were plated at the same density, the SH-SY5Y cells did not grow as well as BE(2)-M17, SJCRH30 or Neuro-2A cells. Therefore, SJCRH30 cells were chosen for further study, because of the increased sensitivity to PbTx-2 at 24 h and because of the large size, which allows for clear pictures and calculations of the morphological parameters.

### 2.2. Cytotoxicity of PbTx-1 and PbTx-2 to SJCRH30 and Neuro-2A Cell Lines

To compare the human cell line model (SJCHR30) to the traditional PbTx cytotoxicity model (murine derived Neuro-2A), we performed comparative cytotoxicity experiments with the XTT assay and the nuclear fluorescent stain Hoechst 33342 (H-dye) microscopy assay. EC_50_ values were calculated from cytotoxicity curves for each experiment (Table 3 and Table 4) and reflect a calculation of at least *n =* 3 separate experiments. Two concentrations of veratridine (25 μM and 50 μM) were used for the Neuro-2A cell line, because of the variability in previous literature. As shown in Table 3 and Table 4, SJCRH30 cell lines show greater sensitivity to PbTxs after 48 h when compared to Neuro-2A cell lines without ouabain and veratridine. As such, we suggest this cell line as a preferable model for cytotoxicity when the goal is to observe the effects of PbTxs in the absence of confounding factors, such as ouabain and veratridine. In addition, there was no significant difference between the H-dye nuclear stain microscopy assay or XTT assay for either SJCHR30 or Neuro-2A cell lines without ouabain and veratridine.

**Table 3:** EC_50_ values for cytotoxicity of PbTx-1 on SJCRH30 and Neuro-2A cells as measured by the XTT and H-dye assays. * indicates significance from SHCRH30, and ^#^ indicates significance from Neuro-2A (values in the same column).

Cell Line	H-Dye: 24 h	H-Dye: 48 h	XTT: 24 h	XTT: 48 h
SJCRH30	2.38 ± 0.69 μM	1.79 ± 0.45 μM	3.23 ± 0.90 μM	2.04 ± 0.07 μM
Neuro-2A	2.80 ± 0.91 μM	2.51 ± 0.25 μM *	3.75 ± 1.16 μM	3.93 ± 1.07 μM *
Neuro-2A (25 μM Veratridine, 500 μM ouabain)	6.69 ± 3.12 nM *^,#^	6.61 ± 1.29 nM *^,#^	10.2 ± 6.57 μM	2.85 ± 1.18 μM
Neuro-2A (50 μM Veratridine, 500 μM ouabain)	2.79 ± 1.24 nM *^,#^	4.74 ± 2.18 nM *^,#^	0.73 ± 1.20 μM *^,#^	0.67 ± 1.06 μM ^#^

**Table 4:** EC_50_ values for cytotoxicity of PbTx-2 on SJCRH30 and Neuro-2A cells as measured by the XTT and H-dye assays. * indicates significance from SHCRH30, and ^#^ indicates significance from Neuro-2A (values in the same column).

Cell Line	H-Dye: 24 h	H-Dye: 48 h	XTT: 24 h	XTT: 48 h
SJCRH30	1.76 ± 0.37 μM	0.52 ± 0.09 μM	2.60 ± 1.33 μM	1.10 ± 0.97 μM
Neuro-2A	2.09 ± 0.65 μM	1.94 ± 0.17 μM *	2.60 ± 0.31 μM	2.83 ± 0.69 μM
Neuro-2A (25 μM veratridine, 500 μM ouabain)	2.40 ± 1.80 μM	0.43 ± 0.68 μM ^#^	6.78 ± 3.30 μM	5.49 ± 0.90 μM *
Neuro-2A (50 μM veratridine, 500 μM ouabain)	20.6 ± 6.23 nM *^,#^	17.9 ± 8.87 nM *^,#^	1.59 ± 1.41 μM	3.99 ± 3.61 μM

**Figure 3:**
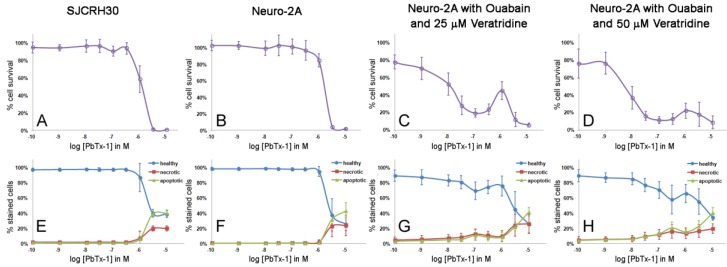
Cytotoxicity and apoptosis assay curves of cells treated with PbTx-1 for 48 h. (**A**–**D**) (purple open circle) the cytotoxicity assay as measured by fluorescent nuclear stain. (**E**–**H**) The apoptosis assay for apoptosis as measured by Annexin V staining (green triangle), necrosis as measured by Annexin V/PI co-staining (red square) and healthy cells as measured by no staining (blue circle). Each point represents the mean ± standard deviation for *n =* 3–5 experiments.

**Figure 4:**
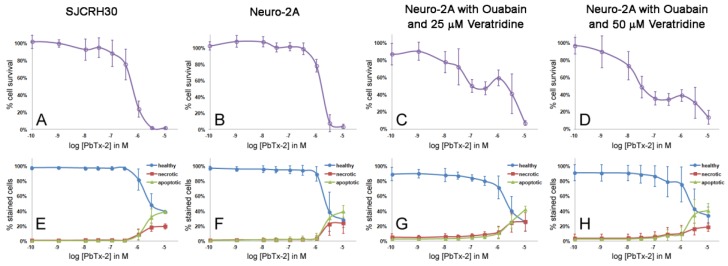
Cytotoxicity and apoptosis assay curves of cells treated with PbTx-2 for 48 h. (**A**–**D**) (purple open circle) the cytotoxicity assay as measured by fluorescent nuclear stain. (**E**–**H**) The apoptosis assay for apoptosis as measured by Annexin V staining (green triangle), necrosis as measured by Annexin V/PI co-staining (red square) and healthy cells as measured by no staining (blue circle). Each point represents the mean ± standard deviation for *n =* 3–5 experiments.

When ouabain and veratridine were added to the Neuro-2A cell cytotoxicity assay, several factors were observed (Table 3 and Table 4). First, there was an increase in sensitivity to PbTxs, although the variability greatly increased, as demonstrated by the large standard deviations for the EC_50_ values. Second, the XTT assay did not always detect this increase in sensitivity. Neuro-2A cells treated with PbTxs, ouabain and 50 μM veratridine showed sensitivity to PbTxs in the nanomolar range when assayed on the H-dye nuclear stain assay, but only the micromolar range when assayed on the XTT assay (Table 3 and Table 4). Ouabain/veratridine-treated Neuro-2A cells did not show a typical sigmoidal dose response curve in response to PbTxs, but rather have a polynomial shaped curve, which made calculating EC_50_ values highly variable at both 24 and 48 h (48 h shown, Figure 3 and Figure 4). This oddly shaped curve was observed in some XTT experiments, but not all, and not to the extreme that could be seen with the H-dye nuclear stain assay. Ouabain/veratridine-treated Neuro-2A cells appeared to show an initial cytotoxicity to PbTxs at 0.1 μM, with recovery around 1 μM and nearly complete toxicity at 10 μM. The percentage of cell death at initial cytotoxicity varied according to the PbTx used, with PbTx-1 yielding 20%–30% and PbTx-2 yielding 40%–50% cytotoxicity at 0.1 μM (Figure 3 and Figure 4). The variation in EC_50_ values observed within the experiments of this study and, between experiments of the literature, could be explained by the highest concentration used to calculate EC_50_ values from corresponding sigmoidal dose response curves. If studies only used up to 0.1 μM PbTx, rather than the highest 10 μM dose used in this study, then complete cytotoxicity might not have been obtained. Furthermore, the traditional MTT or XTT assay might not even be able to detect the recovery seen at 1 μM, as the XTT assay was not as sensitive as the H-dye nuclear stain assay in our study.

### 2.3. Apoptosis/Necrosis Effects of PbTx and/or Ouabain and Veratridine

To investigate the causes of the cytotoxicity recovery seen at 1 μM PbTx, SJCRH30 and Neuro-2A (with and without ouabain and veratridine) were stained with Annexin V and propidium iodide (PI). Annexin V is a protein that transitions to the outside of cell membranes during apoptosis. Cells stained for Annexin V only are considered apoptotic. Cells stained for both Annexin V and PI are considered necrotic, as the cell membranes become permeable during necrosis and the stains can enter the cell [32,33].

Without the presence of ouabain and veratridine, apoptosis and necrosis events were low for both SJCRH30 and Neuro-2A cells in the presence of PbTx-1 or PbTx-2 until concentrations of 5–10 μM (Figure 3E,F and Figure 4E,F). Apoptosis was significantly higher than necrosis for SJCHR30 cells with 5 μM PbTx-1 and 10 μM PbTx-1 and PbTx-2. The increase in apoptosis and necrosis for SJCRH30 and Neuro-2A cells at 5–10 μM PbTxs corresponded to the same dose at which the cytotoxicity assay measured 0% cell survival (Figure 3A,B and Figure 4A,B).

With the presence of ouabain and veratridine, Neuro-2A cells exhibited an initial decline in cell survival, followed by recovery at 1 μM PbTxs and, then, a second decline in cells survival to the maximal dose of PbTxs (Figure 3C,D and Figure 4C,D). Similar triphasic response patterns in cytotoxicity were also seen at 24 h treatment (data not shown). Apoptosis and necrosis events were higher (3%–4%) in ouabain and veratridine-treated cells than untreated cells (0%–1%) at low to zero presence of PbTxs, likely due to the presence of these neurotoxins. Apoptosis and necrosis in ouabain, veratridine and PbTx-treated cells increased slightly around 0.1 μM PbTxs before declining again at 1 μM, corresponding to the dose of initial decline and recovery in cell survival exhibited in cytotoxicity assays. This effect was seen most obviously at 50 μM veratridine and PbTx-1 treatment (Figure 3H). While not in perfect alignment with the cytotoxicity assay, the results of the apoptosis assay indicate that some cellular factor(s) may be reducing apoptosis and/or necrosis in Neuro-2A cells treated with ouabain, veratridine and PbTxs around a 1-μM concentration.

Triphasic apoptotic responses, such as those seen in this study, have previously been documented in irradiated zebrafish embryos. Below a certain radioactive dose, there was an increase in apoptosis, with hormesis observed at an intermediate dose and increasing apoptosis at high doses above the hormetic dose range [34]. Biphasic hormesis responses to toxins are well known, where high doses are toxic and a low dose has a protective response by initiating detoxifying cellular machinery [35]. While we do not know at this point what causes the hormesis seen with PbTx exposure to ouabain/veratridine-treated Neuro-2A cells, it is likely due to the activation of detoxifying protective systems within the cells. The width of the hormetic dose range in toxicology is typically less than 100-fold in dose [36], which is consistent with PbTx hormesis in our study.

### 2.4. Cell Morphology of SJCRH30 and Neuro-2A Cell Lines

**Figure 5:**
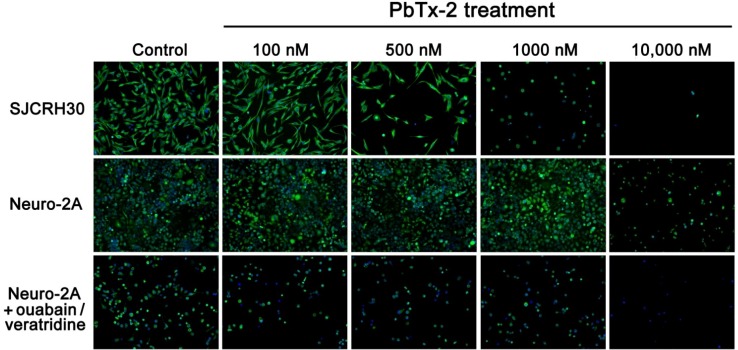
Cell morphology at increasing doses of PbTx-2 after 48 h. Cells were stained with Hoechst 33342 nuclear dye (blue) and Tubulin Tracker (green). A representative experiment is shown.

In addition to the increased efficiency and sensitivity of live cell counting associated with the H-dye nuclear stain assay, the microscopy method is also able to detect other morphological parameters of cell lines depending on what parts of the cell are stained fluorescently. As shown in Figure 5, cells can be visually seen in decreasing numbers as PbTx-2 concentrations increased. In addition, the SJCRH30 cells are most observant in changing shape, with a less spread out morphology (indicated by less tubulin) by 1000 nM PbTx-2. The lower sensitivity of Neuro2A cells to PbTx-2 as compared to SJCRH30 cells, reflected in the higher EC_50_ values of Table 4, can be visually seen in Figure 5. In addition, the morphological changes effected by ouabain and veratridine, in addition to the decrease in live cells, due to ouabain and veratridine (reflected by Table 2), can also be seen by the fluorescence microscopy assay (Figure 5).

The Image Xpress Micro and corresponding MetaXpress Image Acquisition and Analysis software (Molecular Devices, Sunnyvale, CA, USA) are also able to calculate several morphological parameters of cells in the cytotoxicity assay. Using the SJCRH30 cell images obtained in Figure 5, average cell area, nuclear area and tubulin area, as well as the percentage of cells with tubulin were calculated and reported in Table 5, along with the cell counts that are used for calculating cytotoxicity curves. This is a representative example of what can be calculated along with cytotoxicity to gain a greater understanding of the effects of PbTxs during cytotoxicity. As shown in Table 5, not only does cell count decrease with increasing concentrations of PbTx-2, but so does the percentage of cells with tubulin, cell area and tubulin area. Both tubulin area and cell area show an increase around 500 nM, indicating cell swelling. This occurs at the concentration corresponding to right before Annexin-V staining (an early indicator of apoptosis) begins to increase in SJCRH30 cells (Figure 4). Interestingly, nuclear area does not decrease with increasing concentrations of PbTx-2 (Table 5).

**Table 5:** SJCRH30 cell morphology data from Figure 5.

[PbTx-2]	Cell Count	Cell Area	Nuclear Area	% Cells with Tubulin	Tubulin Area
0	289	637.8	203.0	95.1%	621.1
100 nM	234	681.2	209.7	98.3%	674.6
500 nM	74	908.6	258.7	94.6%	899.2
1000 nM	50	367.1	222.4	74.0%	331.5
10,000 nM	3	352.5	251.7	66.7%	290.1

## 3. Experimental Section

PbTx-1 and PbTx-2 were extracted and purified from *K*.* brevis* (Wilson strain) unialgal cultures, as previously described [37,38,39]. Ouabain and veratridine were purchased from Sigma-Aldrich (St. Louis, MO, USA). CELLCOAT poly-d-lysine 96-well tissue culture plates were purchased from Greiner BioOne (Monroe, NC, USA). Phosphate buffered saline (PBS), Hanks Buffer Saline Solution (HBSS), penicillin, streptomycin, fetal bovine serum (FBS), horse serum, Tubulin Tracker 488, Hoechst 33342 (H-dye), Annexin-V-Alexa Fluor 488 and propidium iodide were purchased from Invitrogen Life Technologies (Grand Isle, NY, USA).

The B35 (CRL-2754), BE(2)-M17 (CRL-2267), Neuro-2A (CCL-131), PC-12 (CRL-1721), SH-SY5Y (CRL-2266) and SJCRH30 (CRL-2061) cell lines were purchased from ATCC (Manassas, VA, USA). The B50 cell line was purchased from Sigma-Aldrich (St. Louis, MO, USA). All cell lines were seeded at 5000 cells per well in a 96-well plate, with the exception of PC-12 cells, which were seeded at 10,000 cells per well, because of their small size. B35 and B50 cells were cultured in DMEM with GlutaMAX (Invitrogen Life Technologies, Grand Isle, NY, USA) containing 10% FBS. BE(2)-M17 and SH-SY5Y cells were cultured in EMEM/F12K (1:1) (ATCC, Manassas, VA, USA) containing 10% FBS. Neuro-2A cells were cultured in EMEM (ATCC, Manassas, VA, USA) containing 10% FBS. SJCRH30 cells were cultured in RPMI (ATCC, Manassas, VA, USA) containing 10% FBS. PC12 cells were cultured in RPMI containing 10% horse serum and 5% FBS (ATCC, Manassas, VA, USA). All cells were incubated at 37 °C for 24 h prior to treatment. Cells were then treated with PbTx-1 or PbTx-2 (0.1 nM to 30 μM). A subset of cells were also treated with ouabain (500 μM) and veratridine (25 μM or 50 μM) at the same times as PbTx treatment. Following 24–72 h of treatment, cells were assayed for cytotoxicity or apoptosis activity.

Cytotoxicity was measured using fluorescence staining of cell nuclei. At the time of the treatment described above, control and treated cell nuclei were stained with 0.1 μg/mL Hoechst 33342 (H-dye) in PBS. Following 24–72 h of treatment, non-adherent cells were removed with the media, and adherent cells were washed with 200 μL pre-warmed HBSS per well. Cells were then stained with 200 nM Tubulin Tracker 488 in HBSS at 37 °C for 20 min. The intensity of fluorescence of the H-dye stain was measured on the DAPI channel of the Image Xpress Micro (Molecular Devices, Sunnyvale, CA, USA) using the MetaXpress Image Acquisition and Analysis v2.0.1.44 software (Molecular Devices, Sunnyvale, CA, USA). The percentage of positively-stained cells as compared to control treatments (percentage of maximum) averaged within 4 snapshots of each well were calculated with SAS v9.1.3 software (SAS Institute Inc., Cary, NC, USA) and analyzed with non-linear regression curve-fit analysis by GraphPad Prism v4.03 (San Diego, CA, USA) to yield EC_50_ values. In addition, pictures of cells from the cytotoxicity experiments stained with H-dye and Tubulin Tracker were visualized using the MetaXpress Image Acquisition and Analysis v2.0.1.44 software (Molecular Devices, Sunnyvale, CA, USA).

The XTT cell proliferation assay (kit from ATCC, Manassas, VA, USA) was performed according to instructions from the manufacturer. Activated-XTT solution was added (50 μL) to each well, and the intensity of color was measured following 1 h of development with absorbance at 475 nm on the Flex Station III microplate reader (Molecular Devices, Sunnyvale, CA, USA) using the SoftMax Pro 5.2 software (Molecular Devices, Sunnyvale, CA, USA). A reference reading was taken at 660 nm to assess non-specific absorbance. The percentage of maximum curves were analyzed with non-linear regression curve-fit analysis by GraphPad Prism v4.03 (GraphPad Software, San Diego, CA, USA) to yield EC_50_ values.

Apoptosis and necrosis of cells was measured using fluorescence staining of Annexin V. 48 h post-treatment as described above, cells were stained with Annexin-V-Alexa Fluor 488 (Invitrogen Life Technologies, Grand Isle, NY, USA) and propidium iodide. The proportion of positive cells for each stain were measured on the Image Xpress Micro and analyzed with SAS v9.1.3 software (SAS Institute Inc, Cary, NC, USA).

EC_50_ values determined for the various experimental setups were compared using ANOVA SAS v9.1.3 software (SAS Institute Inc., Cary, NC, USA). In all experiments, results are presented as the mean ± SD and were considered statistically significant if a *p-*value of less than 0.05.

## 4. Conclusions

The traditional cytotoxicity assay for the detection of PbTxs uses the Neuro-2A cell line, which must first be treated with the neurotoxins, ouabain and veratridine, in order to become sensitive to PbTxs. However, we have demonstrated that a hormesis effect can be seen in this model due to a triphasic dose response to PbTx treatment on ouabain/veratridine-treated Neuro-2A cells at 24–48 h. The triphasic dose response yielded a polynomial shaped curve rather than the typical sigmoidal dose response curve, which made calculating EC_50_ values highly variable. Because of this, great care must be taken when using the ouabain/veratridine-treated Neuro-2A cytotoxicity model for testing samples for the presence of PbTxs.

In this study, we have described a new fluorescence microscopy method, which allows for accurate calculation of cytotoxicity via nuclear staining. This method allowed for the observation of the hormetic effects of PbTxs on ouabain/veratridine-treated Neuro-2A cells, which was largely overlooked with the traditionally used XTT method. In addition, the fluorescence microscopy method allows for additional measurement of other viability parameters depending on which aspect of the cell is stained. We propose using the SJCRH30 cell line as an alternative to Neuro-2A cells, because of the good sensitivity to PbTxs at 48 h without the need for ouabain or veratridine and because their large size allows for clear visualization of the cytotoxicity. It is important to note that testing with tissue extracts and other samples would be needed to ultimately determine the extent that this assay can be used to survey for PbTxs, and ongoing work in our laboratory is directed to this end.
